# Determination of bismuth in environmental samples by slurry sampling graphite furnace atomic absorption spectrometry using combined chemical modifiers

**DOI:** 10.1007/s10661-014-4125-7

**Published:** 2014-11-12

**Authors:** Ryszard Dobrowolski, Joanna Dobrzyńska, Barbara Gawrońska

**Affiliations:** Department of Analytical Chemistry and Instrumental Analysis, Faculty of Chemistry, Maria Curie-Sklodowska University, M. C. Sklodowska Sq. 3, 20-031 Lublin, Poland

**Keywords:** Bismuth determination, Permanent modifiers, Soil and sediment, Slurry sampling graphite furnace atomic absorption spectrometry

## Abstract

Slurry sampling graphite furnace atomic absorption spectrometry technique was applied for the determination of Bi in environmental samples. The study focused on the effect of Zr, Ti, Nb and W carbides, as permanent modifiers, on the Bi signal. Because of its highest thermal and chemical stability and ability to substantially increase Bi signal, NbC was chosen as the most effective modifier. The temperature programme applied for Bi determination was optimized based on the pyrolysis and atomization curves obtained for slurries prepared from certified reference materials (CRMs) of the soil and sediments. To overcome interferences caused by sulfur compounds, Ba(NO_3_)_2_ was used as a chemical modifier. Calibration was performed using the aqueous standard solutions. The analysis of the CRMs confirmed the reliability of the proposed analytical method. The characteristic mass for Bi was determined to be 16 pg with the detection limit of 50 ng/g for the optimized procedure at the 5 % (*w*/*v*) slurry concentration.

## Introduction

Recently, the use of Bi in various industries has been growing rapidly because of its specific chemical and physical properties. It is frequently used in medicine, cosmetic industry, semiconductors, alloys, metallurgical additives and preparation of uranium nuclear fuels. Due to the wide application of Bi, its content in the environmental components and the potential for human exposure has permanently increased. According to the World Health Organization, Bi is not an essential element for humans. At present, there is a growing number of evidences for the toxic effects of Bi for people, animals and plants (Pamphlett et al. 2000; Magalhaes et al. 2003). It was confirmed that following an oral intake of Bi compounds, the element enters into the nervous system and damages motor neurons. In chronic exposure, Bi causes nephropathy, osteoarthropathy and hepatitis (Slikkerveer and de Wolf 1989). However, the mobility of Bi in the environment is limited due to relatively low solubility of its compounds. There are numerous techniques applicable for Bi determination, but most of them have some limitations, especially for the analysis of solid environmental samples. Inductively coupled plasma mass spectrometry (ICP MS), inductively coupled plasma optical emission spectrometry (ICP OES) (Hasssanien and Ali 2012; Ivanova et al. 2001; Aulinger et al. 2002; Marques et al. 2000; Krishna and Arunachalam 2004; Gundersen et al. 2000) and the techniques based on the atomic absorption (Ivanova et al. 1997; Sengupta and Bouvier 1995; Moscoso-Perez et al. 2003; Kula et al. 2009) are the most often used for Bi determination. If the multi-elemental analysis is not necessary, the atomic absorption spectrometry (AAS) can be considered the method of choice (Das et al. 2006). Among the above-mentioned techniques, the hydride generation (HG) AAS is the most often used for Bi determination. Even though this analytical approach allows for the on-line Bi preconcentration (Moscoso-Perez et al. 2003; Kratzer and Dedina 2008; Cankur et al. 2002), the use of hydrides generation techniques usually requires complete decomposition of the solid samples prior to analysis, which increases the risk of the sample contamination and prolongs the time of analysis. There is a method in existence where BiH_3_ is generated directly from slurries (Matusiewicz and Sturgeon 2012); however, a quantitative Bi signal is assured only if the finest particles are used and the quantitative extraction of Bi to liquid is reached. The above-mentioned limitations can be overcome by the application of the slurry sampling graphite furnace atomic absorption spectrometry (GFAAS) technique. By applying concentrated slurries and using proper chemical modifiers, the limit of detection for Bi determination by this technique seems to be comparable to this of HG AAS or even lower. Moreover, this technique ensures the simplicity of the preparation, the reduction of the analysis time, minimizes the sample contamination risk and the potential loss of analyte. To the best knowledge of the authors, the slurry sampling GF AAS technique for Bi determination has not been yet proposed, probably because of the typical strong interferences and methodological problems dealing with effective way to eliminate them. One can expect that these limitations can be overcome by applying combined chemical modifiers. There are a few chemical modifiers proposed for Bi determination in the solutions (Elsherif and Kuss 2012; Freschi et al. 2012; Acar et al. 1998; Acar et al. 1997; Barbosa et al. 2001). Among those studied, Pd + Rh + Pt and W + Pd + tartaric acid were the most effective for Bi determination in digested geological samples (Acar et al. 1998; Acar et al. 1997); however, in these cases, the characteristic mass for Bi was very poor. It is noteworthy that the action of the chemical modifiers in slurry sampling GF AAS seems to be more complex than in aqueous solutions, because of the strong interaction of unchanged matrix components with Bi species.

In this paper the combination of permanent and classical modifiers has been studied for Bi determination in the environmental samples by application of the slurry sampling GF AAS technique.

## Experimental

### Instrumentation

AAS-3 (Carl Zeiss, Jena, Germany) atomic absorption spectrometer with a deuterium lamp background correction system, supplied with EA 3 electrothermal atomizer and MPE autosampler, was applied. A Bi hollow cathode lamp (Photron, Narre Warren, Australia) was operated at 10 mA; the analytical line at 223.1 nm was used in the measurements with the spectral bandwidth of 0.2 nm. The volume of the injected sample was 20 μL. The heating program applied for Bi determination is given in Table 1. All measurements were carried out with at least five replicates using pyrolytically coated graphite tubes equipped with pyrolytically coated Lvov platforms obtained from PerkinElmer. Pure argon (99.999 % pure, Air Products, Warsaw, Poland) was used as the purge gas at 280 mL/min, except the atomization stage. Background-corrected integrated absorbance was used as the analytical signal with the integrated time of 5 s.

**Table 1:** Temperature programme used for the determination of Bi in environmental samples by the slurry sampling GF AAS technique

Stage	Temperature [°C]	Ramp/hold [s]
Drying I	120	20/3
Drying II	160	2/40
Pyrolysis	600	200/5
Cooling	120	NP/15
Atomization	2300	1000/5
Cleaning	2600	1000/3

*NP* no power—lack of ramp

### Materials, reagents and solutions

In the all analytical work, high purity deionized water obtained from a Milli-Q water purification system (Billerica, MA, USA) was used. Nitric acid Suprapur (65 %) for the preparation of standard solutions and slurries was purchased from Merck (Darmstadt, Germany). Zirconium (Merck, Darmstadt, Germany), niobium (CPI International, Santa Rosa, USA) and titanium (Merck, Darmstadt, Germany) standard solutions diluted in 5 % (*v/v*) nitric acid and tungsten standard solution (Fluka, Buchs SG, Switzerland) diluted in water were used for graphite platforms modification. Calibration standards were prepared by dilution of the bismuth standard solution (Merck, Darmstadt, Germany) with 5 % (*v/v*) nitric acid. Potassium iodide, sodium chloride, potassium hydrogen sulfate, aluminium standard solution and silicate ions standard solution were obtained from POCH (Gliwice, Poland). Barium nitrate was purchased from Aldrich (Saint Louis, USA).

Lake sediments certified reference materials WQB-1 and WQB-3 were obtained from the National Water Research Institute (Gatineau, QC, Canada). NCSDC73323 coming from LGC Standards (Teddington, UK) and GBW 07302 Stream Sediment purchased from the National Research Center for certified reference materials (CRMs) (Beijing, China) were used as soil- and sediment-certified reference materials.

Mine wastes were collected from Zloty Stok in the southwest Poland. The fly ash was obtained from the heat and power station Wrotkow in Lublin which is supplied with coal from the Lublin Coal Basin.

Transferpette micropipettes (Brand, Wertheim, Germany), a Sartorius R-200D balance (Gottingen, Germany), a vortex agitator and a MPW-50 separator (Precise Mechanics, Warsaw, Poland) were used for the preparation of the standard solutions and slurries. Prior to the slurry preparation, soil, ash, mine waste and sediment samples were ground using a MM-2 vibrational mill (K. Retsch, Haan, Germany), equipped with the tungsten carbide balls and chambers. The effectiveness of grinding was examined by using scanning electron microscopy. It was observed that after 20 min of grinding, about 90 % of the particles did not exceed sizes of 10 μm. The powdered samples were dried at 105 °C in a laboratory oven to constant weight. The slurries were prepared by weighing powdered samples in the Eppendorf’s polyethylene vessels adding 2 mL of 5 % nitric acid and shaking the mixture for 2 min. Before each measurement, in order to reduce the error caused by particle settling, the slurries were homogenized using a vortex agitator.

### Procedure of Lvov platform modification

The modification of pyrolytically coated graphite platforms was carried out by the injection of 20 μL 0.05–1 g/L modifier solution onto the Lvov platform. For appropriate modifier deposition, the platforms were subjected to a temperature programme presented in the previous work (Dobrowolski 2002). Then the recommended temperature programme was applied a few times until the signal for 2 ng Bi was repeatable.

## Results and discussion

### Optimization of the permanent modifier mass

**Fig. 1:**
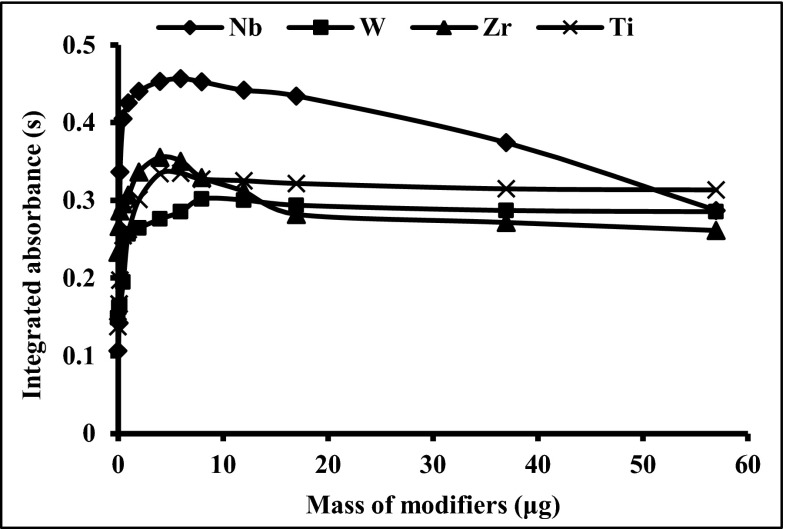
The influence of different modifier masses on the Bi signal (2 ng) at pyrolysis temperature 800 °C, atomization temperature 2500 °C

Optimization of permanent modifier masses was carried out by studying their impact on the analytical signal of Bi, corresponding to the increasing masses of Nb, W, Zr and Ti. The effect of increasing masses of Nb, W, Zr and Ti on the analytical signal of Bi is presented in Fig. 1. With a small change in mass of each modifier, a rapid increase of Bi integrated absorbance is observed. In the case of the platforms modified by NbC, the integrated absorbance for 2 ng of Bi increased significantly, from 0.10 s for unmodified platforms to 0.45 s for that modified by 6 μg of Nb. As presented in Fig. 1 the maximum of integrated absorbance for Bi is obtained for the platforms modified by 6 μg Nb. The integrated absorbance for 2 ng Bi increases rapidly with very small rises in masses of Nb up to 0.5 μg, then increases steadily up to 6 μg, followed by decreases up to 57 μg, to a value of 0.3 s. The use of W, Zr and Ti carbides, as permanent modifiers, also results in the significant increase of Bi integrated absorbance. The integrated absorbance for 2 ng of Bi with respect to the increasing mass of W reaches maximum at around 8 μg, but then with further increases of W mass, it does not change effectively. Comparing the efficiency of W and Nb carbides as permanent modifiers, it was observed that the signal increase for 2 ng Bi obtained for the platform coated by 6 μg of Nb was twice as high as the signal for the platform modified by 8 μg of W. Previously (Dobrowolski 2002), it was stated that NbC formed on the graphite surface is stable at a temperature up to 2500 °C and does not convert into Nb_2_C contrary to W_2_C formation. Based on the above-mentioned insight, one can assume that the action of NbC compared to WC and W_2_C as permanent modifiers should be more predictable and efficient in the case of Bi signal stabilization.

In case of Zr and Ti carbides as permanent modifiers, the maximum of the integrated absorbance for 2 ng Bi is observed for 4 and 6 μg of modifiers, respectively. The further increase of the mass of Ti, up to 60 μg, similarly to W, does not result in the change of Bi absorbance. By contrast, the modification of the platform by Zr in the mass range between 4 and 17 μg leads to decrease of Bi absorbance, while for Zr mass in the range from 17 to 60 μg of Bi integrated absorbance does not change.

On the basis of the results above 6 μg of Nb, 8 μg W, 4 μg Zr and 6 μg Ti have been found to be the most efficient masses of the studied permanent modifiers for Bi stabilization. Taking into account the most efficient action of above-described permanent modifiers, Nb was chosen as the most effective and chemically stable.

### Optimization of the temperature program

**Fig. 2:**
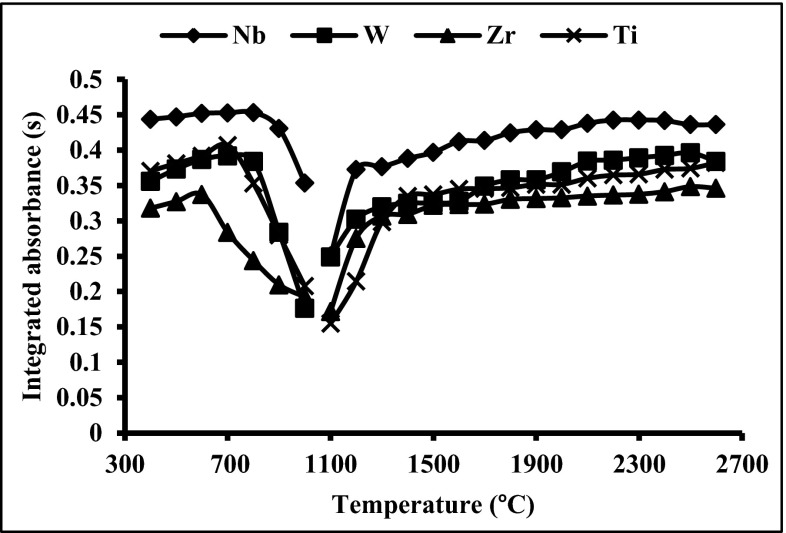
Pyrolysis and atomization curves for 2 ng of Bi using the graphite platforms modified by NbC (6 μg of Nb), WC + W_2_C (8 μg of W), ZrC (4 μg of Zr) and TiC (6 μg of Ti), in aqueous solution; pyrolysis temperature 600–800 °C, atomization temperature 2500 °C

The action of permanent modifiers for the slurry sampling GF AAS technique is much more complicated than for aqueous solutions (Volynsky 2003). Due to the presence of the complex matrix for the sediment and soil samples introduced as slurries onto the graphite platform, different reactions occur between the analyte and matrices which is a cause of interferences. For soil and sediment matrices, the strong impact of silica on the surface of the graphite platform at a temperature higher than 2500 °C can be expected (Dobrowolski 1996). It was proved (Lopez-Garcia et al. 1993; López-García et al. 1996) that the use of concentrated hydrofluoric acid, as liquid medium, for slurries preparation allows to avoid atomizer deterioration and reduce the impact of silica on the analytical signal. However, due to the harmfulness of the concentrated hydrofluoric acid, the protection of the graphite surface by permanent modifiers seems to be more reasonable solution for analysis of this type of materials. Since the most effective removal of matrix interferences occurs at the highest possible temperatures, at which the analyte still remains stable, the studies concerning the effect of the temperature on the stability of Bi on the platforms modified by 6 μg of Nb, 8 μg of W, 4 μg of Zr and 6 of μg Ti, as suggested earlier, were carried out. The pyrolysis and atomization curves for 2 ng Bi in aqueous solution are shown in Fig. 2. Based on the presented relations, it was confirmed that the use of NbC as a permanent modifier provides the highest Bi integrated absorbance, which is in excellent agreement with the observations in the previous section.

It was determined in the comparison of the thermal stability of the Bi signal in the range of the atomization temperatures between 1800 and 2500 °C that the platforms modified by 6 μg of Nb and 4 μg of Zr provide the highest stability. For the platforms modified by 8 μg W and 6 μg Ti, a slight decrease of the analytical signal value, about 12 and 10 %, respectively, was observed in the temperature range from 1800 to 2500 °C. Additionally, it was experimentally documented that for the platforms modified by W carbides at atomization temperatures higher than 2400 °C, double maxima for the Bi signal occur, which could be caused by formation of different tungsten carbides on the graphite surface (Dobrowolski 2002).

It is worth noting that in the case of the platforms modified by W, Zr and Ti carbides, the integrated absorbance of Bi strongly depends on the temperature of pyrolysis. Consequently, it will be difficult to find the optimal temperature of pyrolysis for above-mentioned modifiers. Moreover, the pyrolysis temperature seems to be a critical parameter in defining the robustness of the method. The optimal pyrolysis temperature can change slightly during the lifetime of the tube/platform. For the platforms modified by NbC, the Bi integrated absorbance is stable up to pyrolysis temperature of 800 °C, but above this temperature, a rapid decrease of absorbance is observed. Due to the widest plateau on the pyrolysis and atomization curves and the highest values of integrated absorbance for 2 ng of Bi, the graphite platforms modified by 6 μg of Nb were chosen for further studies.

**Fig. 3:**
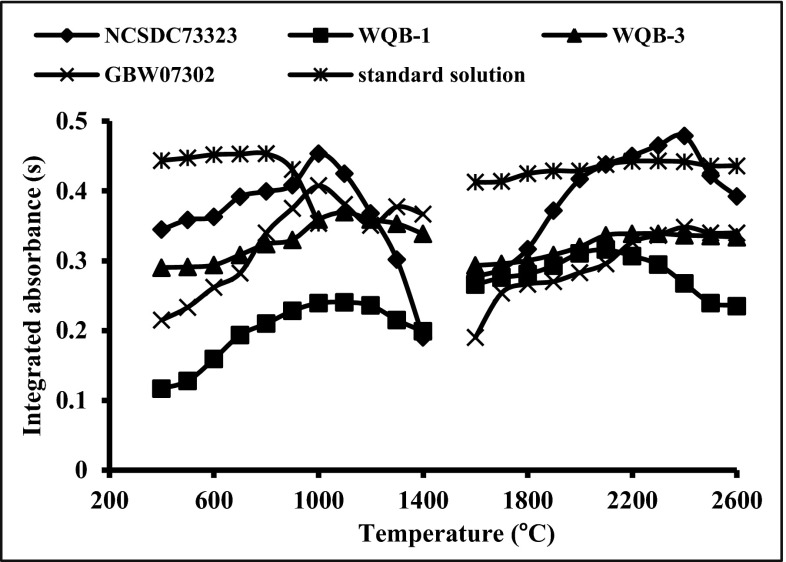
Pyrolysis and atomization curves for 2 ng of Bi using the graphite platforms modified by NbC (6 μg of Nb), in aqueous solution as well as soil and sediment slurries; pyrolysis temperature 800 °C, atomization temperature 2500 °C

To compare the influence of the sediment and soil matrices on the Bi signal, the pyrolysis and atomization curves for 2 ng of Bi, for both the slurries and aqueous solution, are shown in Fig. 3. It was found that pyrolysis and atomization curves of the slurries strongly depend on matrix components both in soils and sediments. The integrated absorbance for Bi in the aqueous solution is stable in the pyrolysis temperature range of 400–800 °C, contrary to the results for the studied slurries, for which the Bi integrated absorbance rises steadily with the increase of the pyrolysis temperature up to 1000–1200 °C.

Considering the atomization curves for 2 ng of Bi obtained for soil and sediment slurries, it is obvious that only the integrated absorbance of the soil CRM is comparable to that of the aqueous solution. The values of Bi integrated absorbance for the sediment slurries are about 50 % lower than that of aqueous solution. This indicates serious interferences that are caused by the studied sediments matrices.

### Influence of matrix components on the analytical signal of Bi

**Fig. 4:**
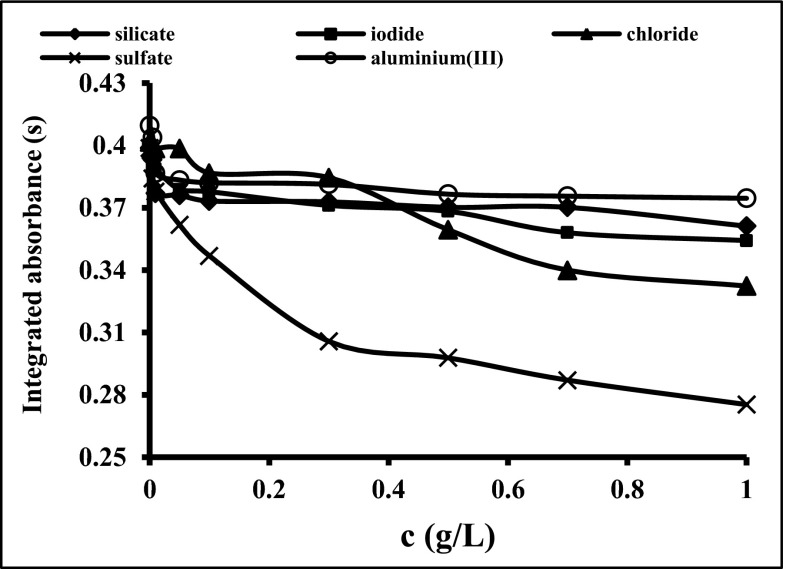
The influence of silicates, iodides, chlorides, sulfates and aluminium (III) ions on 2 ng Bi signal

**Fig. 5:**
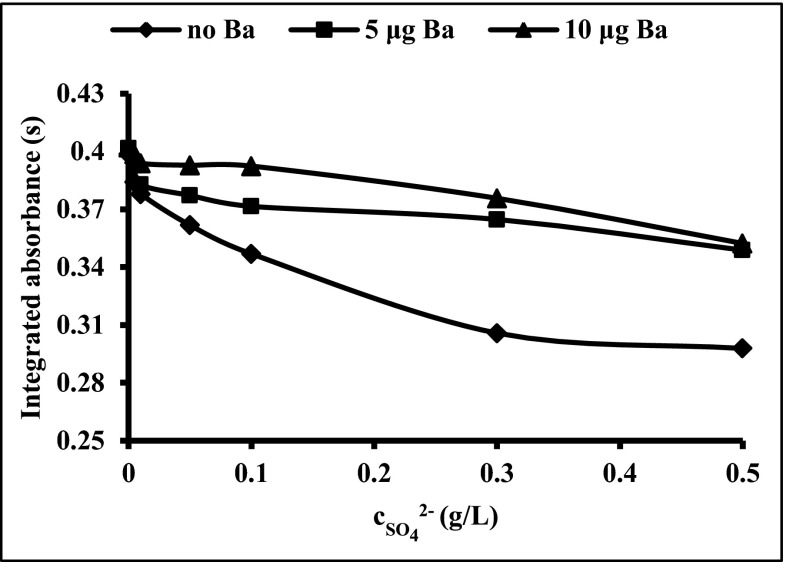
The influence of barium on 2 ng Bi signal in the presence of sulfates

**Fig. 6:**
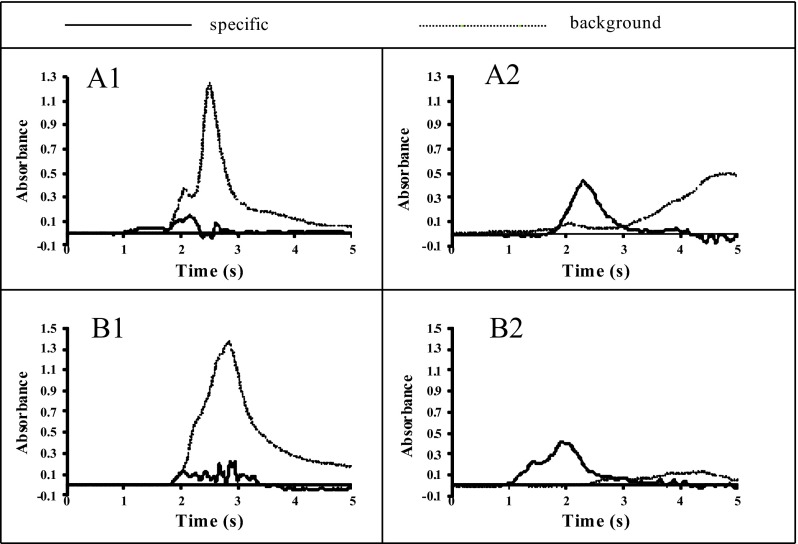
Absorbance-time profiles and background for the atomization of fly ash (**a**) and NCSDC 73323 (**b**) slurries using unmodified graphite platform (*1*) and in the presence of NbC and Ba(NO_3_)_2_ (*2*). Pyrolysis temperature 800 °C, atomization temperature 2500 °C

Due to the fact that the values of Bi integrated absorbance obtained for slurries of the soils and sediments were mostly suppressed (see Fig. 3), searching of the source of interferences was essential. In order to estimate the impact of major matrix components on the Bi signal, some modeling studies were carried out. By evaluating the matrix components of the studied certified reference materials, it was established that alumina, chlorides, iodides, sulfates and silicates are expected to be the source of interference. Taking into account the content of these species in typical slurries, the concentration range of potential interferents was selected. The Bi integrated absorbance in respect to the concentration of the above-mentioned interferents was studied in detail using platforms modified by NbC as presented in Fig. 4. An excess of iodides, Al(III) ions and silicates decreases the value of Bi signal at about 10 % for the concentration of interfering ion at 1 g/L. A slightly greater effect on the Bi signal is observed for chlorides, which decrease the signal by about 18 % for 1 g/L chloride solution. The strongest interference is observed in case of sulfates, where the value of Bi signal decreases steadily with an increase of SO_4_^2−^ concentration. The presence of sulfates at the concentration of 1 g/L reduces the value of Bi absorbance from 0.40 to 0.27. The Bi signals for slurries prepared from CRMs: GBW07302, WQB-1 and WQB-3 were much lower than was expected (see Fig. 3). The preliminary studies of these CRMs by XRF technique confirmed the content of sulfur up to 0.8 %. For this reason, the use of Ba(NO_3_)_2_ as a chemical modifier was proposed. The action of this modifier is based on the creation of insoluble and thermally stable BaSO_4_. Formation of BaSO_4_ results in greater thermal stabilization of sulfur species up to the temperature of its thermal decomposition (Volynsky 2003). In Fig. 5, the influence of different masses of Ba on the Bi signal in the presence of sulfates is shown. The addition of 10 μg of Ba to the graphite platform effectively eliminates interferences originating from sulfates, providing their concentration in the aqueous sample does not exceed 0.1 g/L. In analytical work, the mass of Ba introduced onto the platform should be optimized, avoiding Ba excess, especially for samples containing a large amount of sulfur. This is important because the excess of Ba causes corrosion of the graphite surface by formation of barium carbides. The use of NbC-modified graphite platforms simultaneously with application of Ba(NO_3_)_2_ as the chemical modifier allows the increase in the pyrolysis temperature and successfully correcting background. The influence of NbC and Ba(NO_3_)_2_ on Bi analytical signal and background profile presented in Fig. 6 confirms the veracity and effectiveness of the proposed modification. Moreover, the application of Ba(NO_3_)_2_ causes separation of the background interference from the specific Bi signal.

### Determination of Bi in environmental samples

Graphite platforms modified by 6 μg of Nb were chosen for Bi determination in the environmental samples by the slurry sampling GF AAS technique. To overcome interferences coming from sulfur species, Ba(NO_3_)_2_ was added. The calibration curve for Bi determination has been assessed with a blank and five aqueous calibration solutions in the concentration range of 10–100 μg/L. A characteristic mass of 16 pg was obtained and the limits of detection and quantification for the proposed method were 50 and 160 ng/g Bi, respectively. The detection and quantification limits were calculated as the average of five blank sample signals plus three or ten times (for LOD and LOQ, respectively) the standard deviation of the signals obtained from five blank samples for the 5 % slurries. The characteristic mass was calculated as an average value of that obtained for all calibration standards. The use of 5 % HNO_3_ as diluent leads to the effective extraction of Bi from CRM slurries into the liquid phase (see Table 2). The yield of Bi extraction depends on the matrix components with the highest extraction value observed for soil. Table 3 shows the final results of Bi determination in CRMs and environmental samples by the slurry sampling GF AAS technique using the modified graphite platforms. The obtained CRM data are in good agreement with certified values, which were confirmed by the method described by Linsinger (2010). The precision of Bi determination by the method presented in this paper can be considered acceptable. The relative standard deviation (RSD%) obtained for five slurries replicates was less than 6 %.

**Table 2:** Analyte partitioning in CRMs slurries prepared in 5 % HNO_3_

CRM	Extraction ratio [%]
NCSDC 73323	86
WQB-1	48
WQB-3	54
GBW 07302	73

**Table 3:** Results of determination of Bi in environmental samples by the slurry sampling GF AAS technique using the graphite platform modified by NbC

Sample	Content of Bi [μg/g]	
	Reference value	Slurry sampling
NCSDC 73323	41 ± 6	39.8 ± 2.1^a^
WQB-1	10.42 ± 0.07^b^	10.40 ± 0.02^a^
WQB-3	7.16 ± 0.15^b^	7.24 ± 0.08^a^
GBW 07302	1.64 ± 0.34	1.91 ± 0.10^a^
Mine wastes	–	309 ± 3.0
Fly ash	–	1.31 ± 0.08

^a^Standard deviation (*n* = 5)

^b^Information value

## Conclusions

The modification of the graphite platforms by Nb, Zr, Ti and W, which form thermally stable carbides, significantly increases the Bi signals. From the studied group of permanent modifiers, NbC is recommended for Bi determination by slurry sampling GF AAS in soils and sediments. The efficiency of Bi atomization is the highest in the presence of NbC; moreover, NbC protects the graphite platform surface against corrosion caused mainly by silicon carbide formation. Sulfur compounds present in stream and lake sediments are the main source of interferences and cause decrease of Bi signals. This interference can be successfully eliminated by the application of Ba(NO_3_)_2_ which reacts with sulfur compounds forming thermally stable BaSO_4_. At the atomization stage, BaSO_4_ is decomposed later than Bi compounds. This behaviour facilitates integration of Bi signals. Recommended modifiers enable application of aqueous standard solutions as a calibration method for Bi determination, even for the concentrated slurries of soils and sediments.

## References

[CR1] Acar O, Turker AR, Kilic Z (1997). Determination of bismuth and lead in geological samples by electrothermal AAS: part 1. Comparative study of tungsten containing chemical modifiers. Fresenius’ Journal of Analytical Chemistry.

[CR2] Acar O, Turker AR, Kilic Z (1998). Determination of bismuth, indium and lead in geological samples by electrothermal AAS: part 2. Comparative study of palladium and molybdenum containing chemical modifiers. Fresenius’ Journal of Analytical Chemistry.

[CR3] Aulinger A, Prange A, Niedergesaess R, Schmolke S, Einax JW (2002). Characterization of elemental pollution and its variations in sediments and suspended particulate matter from the River Elbe via multi-element analysis combined with chemometric data evaluation. Journal of Environmental Monitoring.

[CR4] Barbosa FJ, Lima EC, Zanao RA, Krug FJ (2001). The use of a W-Rh permanent modifier for direct determination of bismuth in urine and whole blood by electrothermal atomic absorption spectrometry. Journal of Analytical Atomic Spectrometry.

[CR5] Cankur O, Ertas¸ N, Ataman OY (2002). Determination of bismuth using on-line preconcentration by trapping on resistively heated W coil and hydride generation atomic absorption spectrometry. Journal of Analytical Atomic Spectrometry.

[CR6] Das AK, Chakraborty R, Cervera ML, de la Guardia M (2006). Analytical techniques for the determination of bismuth in solid environmental samples. Trends in Analytical Chemistry.

[CR7] Dobrowolski R (1996). Determination of Ni and Cr in soils by slurry graphite furnace atomic absorption spectrometry. Spectrochimica Acta Part B.

[CR8] Dobrowolski R (2002). Slurry sampling for the determination of thallium in soils and sediments by graphite furnace atomic absorption spectrometry. Analytical and Bioanalytical Chemistry.

[CR9] Elsherif KM, Kuss HM (2012). Simultaneous multi-element determination of bismuth (Bi), antimony (Sb) and selenium (Se). Advances in Applied Science Research.

[CR10] Freschi GPG, Fortunato FM, Freschi CD, Neto JAG (2012). Simultaneous and direct determination of As, Bi, Pb, Sb and Se and Co, Cr, Cu, Fe, and Mn in milk by electrothermal atomic absorption spectrometry. Food Analytical Methods.

[CR11] Gundersen V, Bechmann IE, Behrens A, Sturup S (2000). Comparative investigation of concentrations of major and trace elements in organic and conventional Danish agricultural crops. 1. Onions (*Allium cepa* Hysam) and peas (*Pisum sativum* ping pong). Journal of Agricultural and Food Chemistry.

[CR12] Hasssanien MM, Ali AZ (2012). Determination of bismuth traces by HG–ICP–OES after separation by cloud point extraction using thiourea and iodide mixture. Arabian Journal for Science and Engineering.

[CR13] Ivanova E, Yan XP, Adams F (1997). Determination of bismuth in cod muscle, lake and river sediment by flow injection on-line sorption preconcentration in a knotted reactor coupled with electrothermal atomic absorption spectrometry. Analytica Chimica Acta.

[CR14] Ivanova J, Djingova R, Korhammer S, Markert B (2001). On the microwave digestion of soils and sediments for determination of lanthanides and some toxic and essential elements by inductively coupled plasma source mass spectrometry. Talanta.

[CR15] Kratzer J, Dedina J (2008). Stibine and bismuthine trapping in quartz tube atomizers for atomic absorption spectrometry — method optimization and analytical applications. Spectrochimica Acta Part B.

[CR16] Krishna MVB, Arunachalam J (2004). Ultrasound-assisted extraction procedure for the fast estimation of major, minor and trace elements in lichen and mussel samples by ICP-MS and ICP-AES. Analytica Chimica Acta.

[CR17] Kula I, Arslan Y, Bakırdere S, Titretir S, Kenduzler E, Ataman OY (2009). Determination and interference studies of bismuth by tungsten trap hydride generation atomic absorption spectrometry. Talanta.

[CR18] Linsinger, T. (2010). ERM application note 1, European Commission—Joint Research Centre Institute for Reference Materials and Measurements (IRMM), Geel.

[CR19] Lopez-Garcia I, Arroyo-Cortéz J, Hernández-Córdoba M (1993). Slurry-electrothermal atomic absorption spectrometry of samples with large amounts of silica. Determination of cadmium, zinc and manganese using fast temperature programmes. Analytica Chimica Acta.

[CR20] López-García I, Sánchez-Merlos M, Hernández-Córdoba M (1996). Rapid determination of selenium in soils and sediments using slurry sampling electrothermal atomic absorption spectrometry. Journal of Analytical Atomic Spectrometry.

[CR21] Magalhaes CG, Nunes BR, Giacomelli MBO, da Silva JBB (2003). Direct determination of bismuth in urine samples by electrothermal atomic absorption spectrometry: study of chemical modifiers. Journal of Analytical Atomic Spectrometry.

[CR22] Marques MJ, Salvador A, Morales-Rubio A, de la Guardia M (2000). Trace element determination in sediments: a comparative study between neutron activation analysis (NAA) and inductively coupled plasma-mass spectrometry (ICP-MS). Microchemical Journal.

[CR23] Matusiewicz H, Sturgeon RE (2012). Chemical vapor generation with slurry sampling: a review of applications to atomic and mass spectrometry. Applied Spectroscopy Reviews.

[CR24] Moscoso-Perez C, Moreda-Pineiro J, Lopez-Mahia P, Muniategui S, Fernandez-Fernandez E, Prada-Rodriguez D (2003). Bismuth determination in environmental samples by hydride generation/electrothermal atomic absorption spectrometry. Talanta.

[CR25] Pamphlett R, Stottenberg M, Rungby J, Danscher G (2000). Uptake of bismuth in motor neurons of mice after single oral doses of bismuth compounds. Neurotoxicology and Teratology.

[CR26] Sengupta JG, Bouvier JL (1995). Direct determination of traces of Ag, Cd, Pb, Bi, Cr, Mn, Co, Ni, Li, Be, Cu and Sb in environmental waters and geological materials by simultaneous multi-element graphite furnace atomic absorption spectrometry with Zeeman-effect background correction. Talanta.

[CR27] Slikkerveer A, de Wolf FA (1989). Pharmacokinetics and toxicity of bismuth compounds. Medical Toxicology and Adverse Drug Experience.

[CR28] Volynsky AB (2003). Chemical modifiers in modern electrothermal atomic absorption spectrometry. Journal of Analytical Chemistry.

